# Oncogenic activation of *EEF1A2* expression: a journey from a putative to an established oncogene

**DOI:** 10.1186/s11658-023-00519-9

**Published:** 2024-01-03

**Authors:** Saket Awadhesbhai Patel, Md. Khurshidul Hassan, Manjusha Dixit

**Affiliations:** 1 School of Biological Sciences, National Institute of Science Education and Research, Room No. 204, P.O. Jatni, Khurda, Bhubaneswar, Odisha 752050 India; 2 Homi Bhabha National Institute, Training School Complex, Anushaktinagar, Mumbai, 400094 India

**Keywords:** Cancer, EEF1A1, EEF1A2, PI3K, AKT

## Abstract

Protein synthesis via translation is a central process involving several essential proteins called translation factors. Although traditionally described as cellular “housekeepers,” multiple studies have now supported that protein initiation and elongation factors regulate cell growth, apoptosis, and tumorigenesis. One such translation factor is eukaryotic elongation factor 1 alpha 2 (EEF1A2), a member of the eukaryotic elongation factor family, which has a canonical role in the delivery of aminoacyl-tRNA to the A-site of the ribosome in a guanosine 5′-triphosphate (GTP)-dependent manner. EEF1A2 differs from its closely related isoform, EEF1A1, in tissue distribution. While EEF1A1 is present ubiquitously, EEF1A2 replaces it in specialized tissues. The reason why certain specialized tissues need to essentially switch EEF1A1 expression altogether with EEF1A2 remains to be answered. Abnormal “switch on” of the *EEF1A2* gene in normal tissues is witnessed and is seen as a cause of oncogenic transformation in a wide variety of solid tumors. This review presents the journey of finding increased expression of *EEF1A2* in multiple cancers, establishing molecular mechanism, and exploring it as a target for cancer therapy. More precisely, we have compiled studies in seven types of cancers that have reported *EEF1A2* overexpression. We have discussed the effect of aberrant *EEF1A2* expression on the oncogenic properties of cells, signaling pathways, and interacting partners of EEF1A2. More importantly, in the last part, we have discussed the unique potential of *EEF1A2* as a therapeutic target. This review article gives an up-to-date account of *EEF1A2* as an oncogene and can draw the attention of the scientific community, attracting more research.

## Introduction

EEF1A1 and EEF1A2 are two isoforms of the EEF1A, which play essential roles in the elongation step of protein translation. *EEF1A2* and *EEF1A1* genes have been mapped to chromosomal positions 20q13.3 and 6q14, respectively. Despite their high degree of similarity in coding regions, they exhibit differences in genetic structure and expression patterns. *EEF1A1* is mostly ubiquitously expressed; in contrast, *EEF1A2* exhibits a more restricted expression pattern, primarily found in differentiated tissues such as skeletal muscle, heart muscle, adrenal gland, oral mucosa, esophagus, seminal vesicle, testis, pancreatic islets, and brain [1, 2]. *EEF1A2* was initially identified as a highly overexpressed gene in 30% of ovarian tumors by Anand et al. [3]. Subsequent studies identified *EEF1A2* as a putative oncogene across various cancers, including breast, liver, gastric, pancreatic, and lung cancers [4]. *EEF1A* was reported as a housekeeping gene, and the findings that housekeeping genes were upregulated, activated, or mutated in carcinomas were dismissed because they do not align with kinase-driven oncogenesis [5]. Further detailed in vitro and in vivo studies established the role of EEF1A2 in imparting oncogenic properties to cells and modulating JAK/STAT, PIP4, AKT, and PI3K/AKT/mTOR signaling pathways [6–14]. *EEF1A2* overexpression has generally been associated with poorer prognosis in pancreatic ductal adenocarcinoma, non-small cell lung carcinoma, and ovarian cancer patients [15–17]. However, in breast cancer, it has been associated with better survival [18]. In addition, EEF1A2 has been demonstrated to serve as an autonomous biomarker for the purpose of risk stratification in the context of prostate cancer [19]. Ever since the multiple roles and widespread overexpression of *EEF1A2* in carcinomas have been identified, efforts have been made to target it for anticancer therapy utilizing miRNAs, natural products, and drugs [20–22].

In this review, we aim to summarize the research findings from 1991 to 2023 on the role of *EEF1A2* as an oncogene across a wide variety of cancers. We will discuss the pathways that are perturbed by abnormal EEF1A2 levels and the role of EEF1A2 in driving the tumorigenesis process. To understand the effect of *EEF1A2* on survival, we will be discussing the correlation of *EEF1A2* levels with the survival rates of patients in different carcinomas. Lastly, we aim to shed some light on the prospects of EEF1A2 as a candidate drug target and summarize the findings to date in this regard.

## Genomic context

**Fig. 1:**
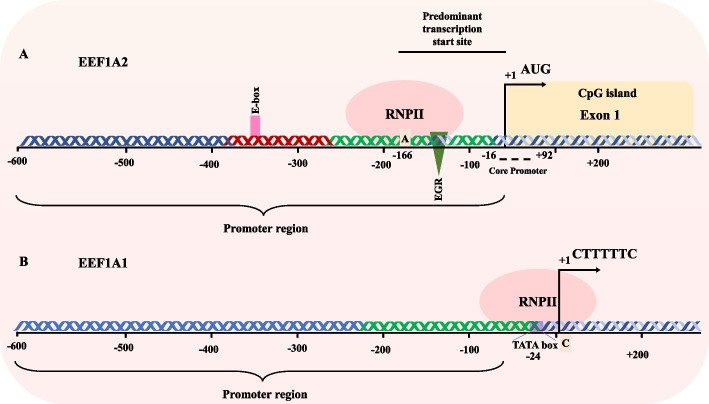
Promoter comparison of both *EEF1A* isoform. **A** Promoter of *EEF1A2* reveals a presence of EGR binding site and E-box. CpG island is present of Exon1. Predominant transcription start site (TSS) is present at −166 position from initiator codon. Core promoter region spans from −16 to + 92 bp. **B** *EEF1A1* promoter discloses a presence of TATA box at −24 position

Using combined fluorescence in situ hybridization (FISH) and PCR analysis, *EEF1A2* and *EEF1A1* were assigned chromosomal positions 20q13.3 and 6q14, respectively [23]. The human *EEF1A2* gene extends nearly 10 kb and comprises eight exons and seven introns. The genomic structure of the *EEF1A2* gene is identical to that of *EEF1A1*, containing the same number of exons as *EEF1A1* (Fig. 1). All positions of intron–exon boundaries are preserved inside the coding region [24]. However, despite the high degree of homology within the coding regions of the two genes, the *EEF1A2* gene is almost thrice as large as *EEF1A1*, owing to larger introns. In addition, there is dissimilarity between the 5′ untranslated region (UTR), 3′ UTR, and upstream promoter elements of the two genes. The predominant transcription start site for *EEF1A2* is located at an adenine residue, 166 bp upstream of the initial AUG codon and resembles the consensus sequence of an Inr element, which possesses a consensus sequence 5′-YYA_+1_N(A/T)YY-3′, where A_+1_ corresponds to the first transcribed base. Nucleotides A_+1_, (A/T)_+3_, and Py_−1_ play a critical role in Inr-mediated transcriptional initiation activity, as emphasized by Smale in 1997. Notably, these particular bases are conserved in the putative initiator of *EEF1A2* [24, 25]. On the other hand, the major transcription start site for the *EEF1A1* gene has been mapped to a cytosine residue present within a stretch of consecutive pyrimidines, a typical feature of ribosomal protein genes. Further analysis reveals that while *EEF1A1* contains a TATA box upstream of the transcription initiation site, *EEF1A2* lacks it [26]. GC analysis of *EEF1A2* gene revealed that the first exon is present within a CpG island. Promoter analysis of the *EEF1A2* gene revealed consensus sequences for important *cis*-regulatory elements, such as an E-box (a crucial regulatory element for muscle-specific genes) and an EGR binding site [24].

## EEF1A1 to EEF1A2: an essential developmental switch

**Fig. 2:**
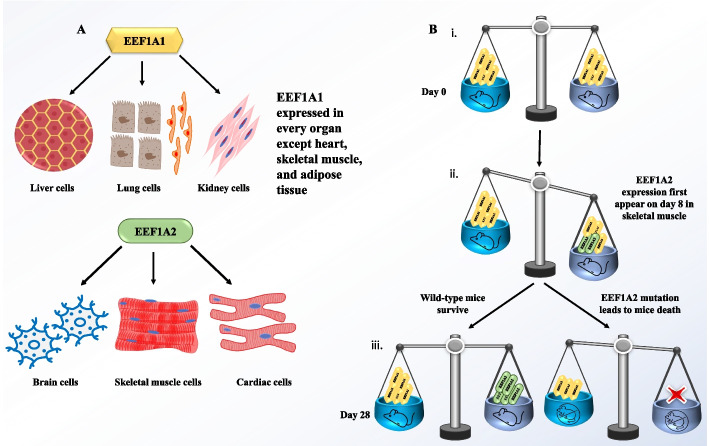
Overview of organ specific switching of *EEF1A1* to *EEF1A2*. **A** *EEF1A1* is expressed in all organs (liver, kidney, lungs, alveolar type1 and type 2 cells) to carry out its canonical functions, except heart, skeletal muscle, and brain where *EEF1A2* expression carries out canonical function. **B** Mice symbol (alive or dead) in weighing dish with cerulean and aegean color represent the specific organs for *EEF1A1* and *EEF1A2* expression. Boxes with yellow or green colors represent *EEF1A1* and *EEF1A2* expression inside specific organs. Red cross sign represents no expression in organs

In 1993, EF-1α2 (or EEF1A2 as it came to be known later) protein was initially identified as a novel variant of EF-1α (or EEF1A1) protein and found to share 75% similarity in the coding regions at the nucleotide level and 96% similarity in the amino-acid sequence with the latter. The expression pattern of EEF1A2 was found to be quite different from EEF1A1 (Fig. 2A). Northern blotting analysis revealed that it was highly expressed exclusively in skeletal muscle, heart muscle, and brain; which are essentially tissues where most cells are fully differentiated with very little or no cell division. EEF1A1, on the other hand, was expressed ubiquitously in the rest of the tissues, with significantly high rates of cell proliferation in liver, lung, and placenta [27]. Another study undertaken in rats showed that reduction in *EF-1α* mRNA levels during brain, heart, and muscle development occurs concomitantly with activation of *S1* (the rat version of EF-1α2) gene expression. It was followed by a terminal differentiation process in the brain, heart, and muscle [28], indicating a developmental regulation of the expression of both isoforms in a tissue-specific manner. The grave consequences of the disruption of this isoform switching became evident when it was identified that the mutation responsible for autosomal recessive wasted (wst) phenotype in mice was essentially a 15.8 kb deletion spanning the promoter region and first noncoding exon of *EEF1A2* gene. These mice exhibit typical features associated with muscle wasting and neurological and immunological abnormalities commencing at 21 days, and eventually leading to the death of the mice at 28 days. Interestingly, this timeline of manifestation of wasted mice symptoms falls in near-perfect synchrony when *EEF1A2* expression takes over from *EEF1A1* in specialized tissues of the heart, brain, and muscle [29]. The exact timing, location, and evolutionary importance of this switch were further explored in subsequent studies in mice [30] and Xenopus [31]. On postnatal day 2, the expression of *EEF1A2* cannot be detected in skeletal muscle. However, there is observable expression of *EEF1A1* in both skeletal and heart muscle tissues. At day 8, both *EEF1A1/2* are expressed in heart and skeletal muscle. However, by day 15 *EEF1A1* is slightly detectable in muscles. At day 21, *EEF1A1* expression is not detectable in skeletal muscle continuing to complete depletion of expression till day 25 from heart too, and *EEF1A2* expression takes over the function of *EEF1A1* in mice [29] and rats [28] (Fig. 2B). Another report in *Xenopus*, reported that *EEF1A2* is also conserved in nonmammalian vertebrate species. Also, EEF1A1 protein is undetectable by adulthood which is posttranscriptionally controlled and *EEF1A1* mRNA remains similar to *EEF1A2* [31].

## EEF1A2 switching on in tumorigenesis

**Fig. 3:**
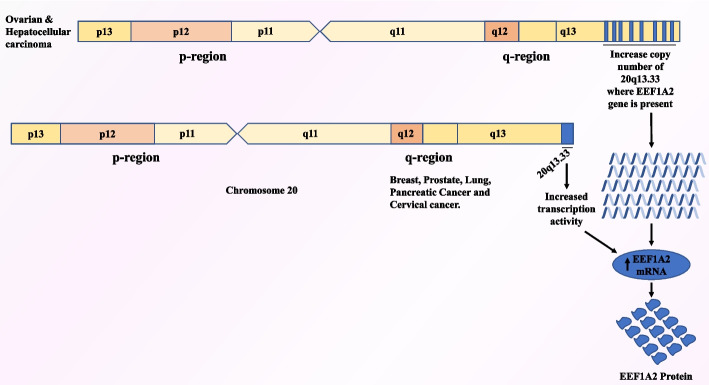
Amplification of *EEF1A2*-containing locus. Chromosome 20 showing increase in 20q13.33 copy numbers or its transcription, enhanced mRNA, and protein level of *EEF1A2* in solid tumors

The pioneering study, which reported that *EEF1A2* is an oncogene and likely to be an important player in the development of cancer, was published in 2002 by Anand et al. [3]. Taking a cue from the fact that there was a frequent increase in copy number of 20q13 locus in 20–30% of ovarian tumors, the authors were able to eventually speculate that overexpression of *EEF1A2*, gene present within this locus, could be crucial in regulating cell transformation and tumorigenesis in ovarian cancer [3] (Fig. 3). However, the authors could only speculate how a translation factor could trigger tumorigenesis. Initially, it was thought that, like eukaryotic initiation factor 4 E (*EIF4E*), *EEF1A2* could also be promoting tumorigenesis by affecting the rate of translation. Previous studies showed that *EEF1A* could bind F-actin and affect cytoskeletal remodeling, which could also be a possible mechanism via which EEF1A2 could promote tumorigenesis [3]. However, subsequent studies made it increasingly clear that this oncogenic isoform, unlike its “non-oncogenic” counterpart EEF1A1, was able to interact with other oncogenes, tumor suppressor genes, membrane lipids, and modulate vital signaling pathways crucial in regulating cell division, apoptosis, migration, and invasion. Some of the key studies in this regard are discussed hereafter.

### Ovarian cancer

**Fig. 4:**
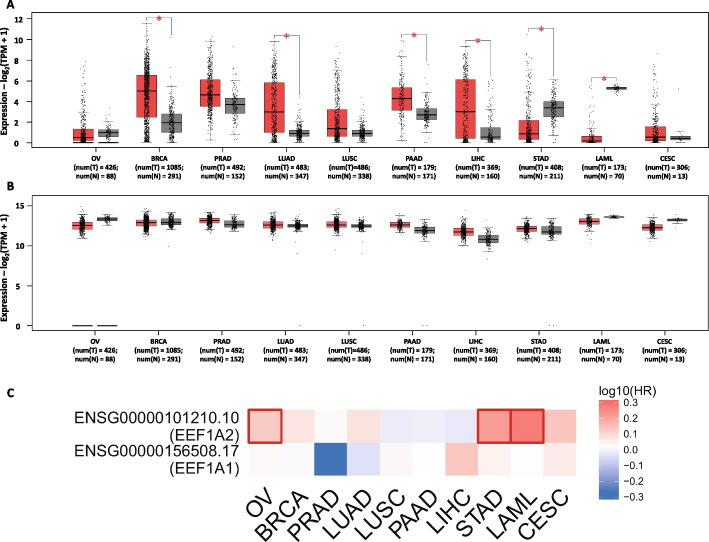
Expression of the *EEF1A1/2* gene and its relationship with patient survival in different cancers. **A**, **B** TCGA datasets shows the expression level of *EEF1A2* (**A**) and *EEF1A1* (**B**) genes in ovarian cancer (OV), breast cancer (BRCA), prostate adenocarcinoma (PRAD), lung adenocarcinoma (LUAD), lung squamous cell carcinoma (LUSC), pancreatic adenocarcinoma (PAAD), liver hepatocellular carcinoma (LIHC), stomach adenocarcinoma (STAD), acute myeloid leukemia (LAML), and cervical squamous cell carcinoma and endocervical adenocarcinoma (CESC). **C** Relationship between *EEF1A2* gene (upper lane) and *EEF1A1* gene (below lane) expression and overall cancer patient survival hazard ratio (HR). *P < 0.05

The role of *EEF1A2* as a putative oncogene was first reported in 2002 in ovarian cancer [3]. Using fluorescence in situ hybridization (FISH), the authors found *EEF1A2* gene amplification in 25% of primary ovarian tumor samples (14 of 53). Using northern blotting, *EEF1A2* mRNA was detectable in 3 of the 11 primary ovarian tumor samples analyzed, but it was absent altogether in normal ovarian tissues. *EEF1A2* mRNA was also present at higher levels in several ovarian carcinoma cells TOV112D, PA-1, HEY, and OV-2008, even though it remained undetected in normal ovarian cell lines NOV-61, OV-90, TOV81D, TOV21G, OVCAR3, OVCAR4, CAOV3, ES-2, or SKOV3. Transcript levels of *EEF1A1* remained constant in normal and carcinoma cell lines. The authors concluded that EEF1A2 expression was elevated in 30% of all tumor tissues and carcinoma cell lines analyzed [3]. Another report examined *EEF1A2* expression at both RNA and protein levels (using isoform-specific antibodies for EEF1A1 and EEF1A2) in histologically defined ovarian tumor subtypes serous, endometrioid, mucinous, and clear cells [32]. The key findings of this study were that a higher proportion of clear cell carcinomas overexpress *EEF1A2* compared with other histological subtypes. Furthermore, the authors noted that *EEF1A2* overexpression is not always a consequence of an increase in copy number. Neither activating mutations in the coding sequence nor methylation of the *EEF1A2* gene were responsible for the elevated transcript levels in the absence of gene amplification event. However, in contrast to the previous study, Tomlinson et al. reported the presence of EEF1A2 in normal ovarian tissue as well [32]. The case for *EEF1A2* overexpression to be selectively associated with clear cell carcinoma histological subtype of ovarian cancer was further substantiated by a recent study showing EEF1A2 to be significantly overexpressed in ovarian clear cells [33]. Exceptionally, the TCGA dataset for ovarian cancer (TCGA–OV) showed a reduction in *EEF1A2* and *EEF1A1* expression compared with GTEx normal tissue (Fig. 4A and B), which could be due to other mutations, sample heterogeneity, normalization techniques or statistical methodologies, sample size, and clinical variations.

High EEF1A2 protein levels predicted an increased 20 year survival probability in serous ovarian tumors [17]. Additionally, analysis of the overall survival plot in the TCGA–OV dataset revealed a high hazard ratio for both the *EEF1A* isoforms in ovarian cancer, as shown in Fig. 4C.

### Breast cancer

Like ovarian cancer, the 20q13.3 locus, which harbors the *EEF1A2* gene, is frequently amplified in breast cancers. Subsequently, Tomlinson and his research team made a significant discovery, finding a remarkable 30-fold increase in the levels of *EEF1A2* mRNA in various types of breast cancer tissues in contrast to healthy breast tissue. Notably, an intriguing trend emerged when considering the Estrogen receptor (ER) status: ER-negative tumors exhibited a modest 1.2-fold rise, whereas ER-positive tumors displayed a substantial 8.4-fold surge in expression compared with normal tissue [34]. The researchers also observed a positive correlation between *EEF1A2* expression and ER positivity. Analyzing EEF1A2 levels through immunohistochemistry on a breast cancer tissue microarray, they found that while normal breast tissue displayed minimal or no presence of EEF1A2 protein, strong and moderate EEF1A2 expression was evident in 11% and 48% of the tumor samples, respectively [34]. However, a preclinical study carried out later with a larger sample size of two independent breast cancer populations including 438 tumor samples inferred that although EEF1A2 protein was detected in 60% of primary breast tumors, there was no significant correlation between EEF1A2 protein levels and ER positivity [18].

**Fig. 5:**
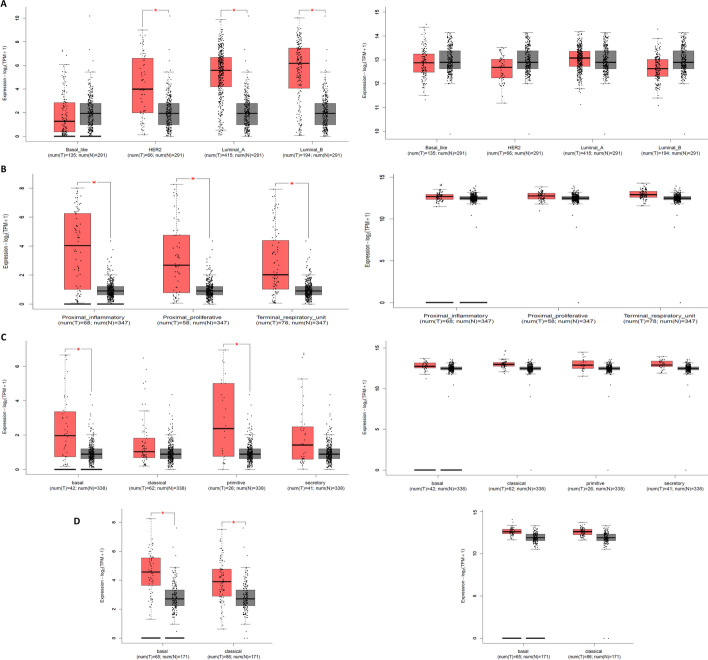
Expression of *EEF1A1/2* gene in molecular subtypes of cancer. **A** Expression level of *EEF1A2* (left) and *EEF1A1* (right) in basal line, HER2, luminal A, and luminal B. **B** Expression levels of *EEF1A2* (left) and *EEF1A1* (right) in proximal inflammatory, proximal proliferative, and terminal respiratory in lung adenocarcinoma. **C** Expression levels of *EEF1A2* (left) and *EEF1A1* (right) in lung squamous cell carcinoma in basal, classical, primitive, and secretory. **D** Expression levels of *EEF1A2* (left) and *EEF1A1* (right) in subtypes of pancreatic adeno carcinoma basal and classical. *P < 0.05

Our investigation of the TCGA database for breast cancer (BRCA) has yielded significant findings regarding the expression of two genes, *EEF1A2* and *EEF1A1*. Specifically, we observed a substantial increase in the expression of *EEF1A2* in BRCA compared with both adjacent normal tissue and GTEx tissue (Fig. 4A). This elevation was consistent across the luminal A, luminal B, and HER2 molecular subtypes (Fig. 5A). However, in the basal subtype, we noticed a decrease in *EEF1A2* expression when compared with adjacent normal tissue and the GTEx normal sample (as depicted in Fig. 5A). In contrast, we did not observe a significant change in the overall expression of *EEF1A1* in BRCA when compared with adjacent normal and GTEx normal tissue (Fig. 4B). Nevertheless, upon analyzing the molecular subtypes separately, we found that the luminal B and HER2 subtypes exhibited a significant reduction in *EEF1A1* expression. Conversely, no significant change was observed in the basal subtype, and the data for the luminal A subtype did not show a significant difference compared with adjacent normal tissue and the GTEx normal sample (Fig. 5A). These findings are consistent with prior reports and align well with the existing literature on the subject.

In instances where tumors lacked lymph node involvement or tested negative for Her2/neu, a notable correlation emerged between moderate to high *EEF1A2* expression levels and enhanced patient survival [18]. The rationale behind the divergent influence of *EEF1A2* on prognosis across various cancer types may potentially stem from its distinct roles within differing tissue contexts or different molecular subtypes. For instance, in the context of triple-negative breast cancer (TNBC), escalated *EEF1A2* levels have been associated with a less favorable prognosis [35]. Notably, this aligns with our scrutiny of TCGA–BRCA data, which unveiled a slightly heightened hazard ratio for *EEF1A2* (Fig. 4C). These findings provide insights into the distinct expression patterns of *EEF1A2* and *EEF1A1* in different molecular subtypes of breast cancer. Further research is warranted to explore the functional implications of these expression changes and their potential role in the development and progression of breast cancer.

### Prostate cancer

Although the initial report implicating EEF1A protein in contributing positively to the proliferation, migration, and invasion of prostate cancer was published in 2011, it was only a year later that the differential role of *EEF1A* isoforms in prostate cancer progression was investigated. Using a panel of tumorigenic (DU145, PC3, LNCaP, 22Rv1) and nontumorigenic (PZHPV7) prostate cell lines with varying rates of cell proliferation and aggressiveness, the authors highlighted the dramatic upregulation of *EEF1A2* but not *EEF1A1* in tumorigenic cell lines [14]. Gene amplification was ruled out as a significant factor for the observed overabundance of EEF1A2 at protein and mRNA levels, suggesting a switching on as a possible mechanism. Furthermore, the authors noted that in comparison to the nontumorigenic cell line PZHPV7, EEF1A2 but not the EEF1A1 protein was specifically enriched in both cytoplasmic and cytoskeletal/nuclear fractions in LNCaP, DU145, and PC3. RNA was extracted from human paraffin-embedded samples in the same study, and *EEF1A2* gene expression was detected in three out of four tumors and all peritumoral hyperplasia samples but not in fresh prostrate benign adenoma samples. *EEF1A1*, on the other hand, was detected in all samples [14]. A subsequent study with a higher sample size (30 pairs of PCa tissues) reported that most of the tumor tissues had elevated *EEF1A2* transcripts and protein levels compared with corresponding normal tissue. However, no clinicopathological correlation was observed with *EEF1A2* levels [36]. The TCGA dataset (PRAD) for prostate adenocarcinoma showed an increase in the expression of *EEF1A2* and *EEF1A1* when compared with adjacent normal tissue and GTEx normal sample, but the difference was not significant (Fig. 4A and B).

Tumors exhibiting a high level of *EEF1A2* expression demonstrated considerably reduced recurrence-free survival, as reported by Worst et al. [19] However, the TCGA dataset (PRAD) displayed an almost negligible hazard ratio for *EEF1A2*, whereas *EEF1A1* had a negative score (Fig. 4C). Drawing any conclusive inference regarding the impact of *EEF1A2* on survival necessitates additional studies and data analysis.

### Lung cancer

*EEF1A2* was initially identified as a putative oncogene in lung cancer using a functional genomics approach. It was found that there is a significant correlation between elevated protein levels and amplification in gene copy number or increased transcript levels of the *EEF1A2* gene in lung adenocarcinoma cell lines (H23, H229, H1792, SK-LU-1, H522, and H1563) [16]. In the same study, immunohistochemistry was performed for 113 patients with stage I non-small cell lung cancer (NSCLC), which confirmed increased EEF1A2 expression in 32 cases (28%) [16]. Another study reported that *EEF1A2* increased epithelial–mesenchymal transformation (EMT) and promoted metastasis in vitro and in vivo in lung adenocarcinoma (LUAD) [37]. In agreement with the observation obtained in lung cancer cell lines, EEF1A2 was expressed in 32% of the cases. The researchers also observed that out of 69 NSCLC patients, 58 cases (84.1%) were EEF1A2 positive. Interesting observations were made by Kawamura et al., where the *EEF1A2* mRNA levels were selectively elevated among the cell lines examined in those of adenocarcinoma (A549, II-18, PC9, H1975, and LCSC) but not of squamous type (LK2). Also, the authors found no significant correlation between *EEF1A2* mRNA expression and protein levels in NSCLC cell lines [38]. The analysis conducted on the TCGA dataset for lung adenocarcinoma (LUAD) and lung squamous cell carcinoma (LUSC) revealed increased levels of *EEF1A2* expression, while no significant change was observed in the expression of *EEF1A1* (Fig. 4A and B). Furthermore, a noteworthy rise in *EEF1A2* expression was observed in proximal proliferative, proximal inflammatory, and terminal respiratory subtypes of LUAD, as well as in basal and primitive types, whereas classical and secretory subtypes of LUSC did not exhibit significant changes (Fig. 5B and C). A difference in the expression patterns of *EEF1A2* in LUAD and LUSC may be due to well-reported clinical, histological, and molecular differences between the two types [39–41]. Furthermore, apart from differences in *EEF1A2* copy numbers, variations in upstream regulators and potential epigenetic modifications within LUAD and LUSC may influence the expression of *EEF1A2*, aspects that require further investigation. Also, no significant changes in *EEF1A1* expression were observed in molecular subtypes of LUAD and LUSC (Fig. 5B and C). In terms of patient survival, overall survival analysis of 113 patients with stage I NSCLC showed that elevated levels of *EEF1A2* is associated with poor prognosis [16]. Molecular subtype LUAD showed a strong positive correlation with *EEF1A2* expression and was associated with poor prognosis [37]. In contrasting, another study showed association of increased *EEF1A2* levels with good prognosis in NSCLC patients. Decreased *EEF1A2* expression showed significant lymph node metastasis and lymphatic invasion [38]. This may be due to mixed samples of LUAD and LUSC. Notably, this corresponds to GEPIA2-based analysis of TCGA dataset where a high hazard ratio was observed for *EEF1A2* in LUAD and a negative hazard ratio for LUSC, similarly *EEF1A1* showed a high hazard ratio for LUSC and a negative hazard ratio for LUAD (Fig. 4C).

### Pancreatic cancer

Immunohistochemistry (IHC) analysis of eEF1A2 staining pattern in pancreatic tissue samples revealed a strong granular cytoplasmic expression in 83% of pancreatic cancer tissues; on the other hand, negative staining was reported for normal pancreatic ducts, chronic pancreatitis samples, and pancreatic islets. mRNA levels of *EEF1A2* were also relatively higher in tissue from pancreatic cancer compared with normal tissue. The same study confirmed the increased levels of *EEF1A2* expression at protein and mRNA levels in pancreatic cancer cell lines (Patu8988, BxPc-3, and Sw1990) [42]. A study on pancreatic ductal carcinoma (PDA) showed 77.8% positive immunoreactivity for EEF1A2, and no expression was observed in normal pancreatic tissue [15]. It is worth noting that EEF1A2 is expressed in normal pancreatic cells, specifically in glucagon-producing pancreatic islet cells [43]. Cao et al. demonstrated very low or no expression of EEF1A2 in normal pancreas and pancreatitis tissue samples [42]. Given that a diabetes susceptibility locus has been mapped to 20q13.3 [43], *EEF1A2* may play a functional role in islet cells. Up until now, studies have predominantly focused on cancers derived from pancreatic ductal epithelium cells, and there have been no reports on *EEF1A2* in cancers originating from glucagon-producing pancreatic islet cells. Consequently, the specific role of *EEF1A2* in cancers derived from glucagon-producing pancreatic islet cells remains unclear.

The TCGA dataset also showed a significant increase in the expression of *EEF1A2* in pancreatic adenocarcinoma (PAAD) and its molecular subtypes, basal and classical, compared with normal tissue and GTEx sample (Figs. 4A and 5D), whereas, *EEF1A1* showed no significant change in the expression when compared with adjacent normal tissue and GTEx normal sample (Figs. 4B and 5D). Also, high expression of *EEF1A2* is associated with poor prognosis [42], but the TCGA dataset (PAAD) from GEPIA2 showed a negative hazard ratio for *EEF1A2* and *EEF1A1* in pancreatic adenocarcinoma (Fig. 4C). As was the case in other cancers, not many studies are available about the effect of *EEF1A2* on the survival of cancer patients.

### Hepatocellular carcinoma

In 2007, a study to prospect the biology of EEF1A proteins in hepatocellular carcinoma (HCC) using cell lines with varying differentiation grades inferred that transcript levels of both *EEF1A* isoforms were increased many fold in undifferentiated cell line JHH6 compared with normal tissue or relatively well-differentiated HuH7, HepG2 cell lines. Increasing mRNA expression with decreasing differentiation grade coincided with an increased nuclear accumulation of eEF1A proteins [44]. Array-based comparative genomic hybridization (CGH) of 67 HCC samples described the commonly amplified chromosomal region 20q13.3 in HCC maps explicitly to a 2.5 Mb region harboring the region *EEF1A2* gene [45]. Furthermore, qRT–PCR analysis of *EEF1A2* mRNA levels in 20 HCC samples and 4 HCC cell lines revealed elevated *EEF1A2* transcript levels in HCC samples compared with normal liver. Likewise, western blot analysis and IHC on tissue microarrays also showed EEF1A2 protein to be moderately or strongly overexpressed selectively in tumor tissues compared with normal liver. Furthermore, no significant difference in *EEF1A2* expression was observed among different etiological groups of HCC such as HBV, HCV, alcoholic, and cryptogenic [45]. In the TCGA dataset of liver hepatocellular carcinoma (LIHC), there was a notable rise in *EEF1A2* expression, while no significant increase was detected in *EEF1A1* expression when compared with adjacent normal tissue and GTEx normal samples (Fig. 4A, B). Intriguingly, the hazard ratio associated with *EEF1A1* exhibited a marked increase while, conversely, a diminished hazard ratio was noted for *EEF1A2* within the same dataset (Fig. 4C). However, it is worth noting that a previous study by Pellegrino et al. demonstrated an association of *EEF1A2* expression with poor prognosis in HCC [12]. These divergent observations might potentially stem from additional mutations inherent to HCC patients, which could diverge across distinct cohorts. Given the paucity of comparable studies, it is imperative to undertake further research to corroborate and authenticate these findings.

### Gastric cancer

Utilizing a population-based tissue microarray constructed from 129 patients who had undergone either radiotherapy or chemotherapy and 24 randomly chosen normal gastric tissues, a pioneer study was undertaken to study the role of *EEF1A2* in gastric cancers [46]. EEF1A2 protein was found to be associated with clinicopathological parameters of gastric carcinoma. Eighty seven patients (67.4%) showed positive lymph node metastasis. *EEF1A2* mRNA levels were also found to be upregulated in a subset of the gastric cancer tissues compared with adjacent normal tissues. Assessment of relative EEF1A2 proteins in 12 paired gastric cancer and corresponding normal mucosa via western blotting confirmed that EEF1A2 protein levels were significantly higher in tumor tissues. This observation was confirmed by IHC of tissue microarray tissues, wherein EEF1A2 protein showed strong immunoreactive staining in 81.40% (105 of 129) of gastric cancer tissues, whereas only 9 out of 24 normal tissues exhibited strong EEF1A2 staining. In general, the EEF1A2 protein displayed a significant upregulation (*P* < 0.001) in gastric cancer tissues, which was associated with an unfavorable prognosis for patients [46]. A recent study conducted in 2023 has demonstrated elevated levels of *EEF1A2* in gastric cancer cell lines. Notably, *EEF1A2* appears to function as an oncogene in the progression of gastric cancer, exerting its influence through the promotion of HSPB8. An intriguing aspect of this research is the observed interaction between EEF1A2 and HSPB8, where *EEF1A2* serves to positively regulate the expression of HSPB8 [47]. A pivotal role for *EEF1A2* emerged in a recent study in gastric cancer revealed that METTL13, which is linked to cancers in humans, works together with *EEF1A2* in a loop that causes abnormal levels of the cancer-promoting gene HN1L [48]. Interestingly, the results from the TCGA dataset (STAD) for stomach adenocarcinoma showed a significant decrease in *EEF1A2* expression compared with adjacent normal tissue and GTEx normal samples (Fig. 4A). This finding contrasts with previous studies, which had suggested a higher expression of *EEF1A2* in cancer cells. However, there was no significant alteration observed in the expression of *EEF1A1* in the STAD dataset (Fig. 4B). Moreover, the hazard ratio analysis in the STAD dataset indicated that *EEF1A2* has a notably high hazard ratio, indicating a potential association with poor prognosis in gastric cancer. On the other hand, there was no significant change in the hazard ratio for *EEF1A1* (Fig. 4C). This study presents a compelling shift in our understanding of the molecular intricacies underlying gastric cancer development, shedding light on the multifaceted roles of *EEF1A2* and HSPB8 in this context. The unexpected findings within the TCGA dataset underscore the complexity of gene expression patterns and their potential implications for cancer progression. Further research is warranted to fully grasp the implications of these novel discoveries and their potential applications in gastric cancer diagnosis and treatment.

### Acute myeloid leukemia

A study in 2020 showed increased expression of *EEF1A2* at mRNA and protein levels. They observed higher *EEF1A2* mRNA and protein levels in acute myeloid leukemia (LAML) cell lines AML-193, Kasumi-1, and KG-1 compared with human normal bone marrow mononuclear cells. Knockout of *EEF1A2* reduced cell proliferation and migration, and increased apoptosis in AML-193 and Kasumi-1, which was rescued by ectopic expression of *EEF1A2*. Mechanistically they found increased dimethylation of eEF1A at lysine 55 in AML-193, Kasumi-1, and KG-1. *EEF1A2* wild-type overexpression stimulated cell proliferation, bolstered migration, and reduced apoptosis. However, the overexpression of eukaryotic translation elongation factor 1 alpha 2 with a K55R substitution did not impact these cellular processes in AML-193 and Kasumi-1 cells. These findings point toward the potential involvement of lysine 55 dimethylation in the oncogenic role of eukaryotic translation elongation factor 1 alpha 2 in acute myeloid leukemia [49]. In the TCGA dataset of acute myeloid leukemia (LAML), there was a significant decrease in *EEF1A2* expression, while no significant decrease was observed in *EEF1A1* compared with normal GTEx samples (Fig. 4A, B). Interestingly, the hazard ratio analysis in the LAML dataset indicated that *EEF1A2* has a notably high hazard ratio, indicating a potential association with poor prognosis in LAML. On the other hand, there was no significant change in the hazard ratio for *EEF1A1* (Fig. 4C). The conflicting findings regarding *EEF1A2* expression in the TCGA dataset compared with the above-mentioned study results raise questions about the expression patterns of *EEF1A2* in cancer progression. To comprehensively grasp the implications of these unique findings and their possible applications in the diagnosis and treatment of leukemia, further in-depth investigation is imperative.

### Cervical cancer

A sole investigation into *EEF1A2* expression in cervical cancer unveiled various alterations among cervical cancer patients, including missense mutations, splice mutations, amplifications, and heightened mRNA levels. Notably, no significant difference was observed in *EEF1A2* gene copy numbers [50]. Interestingly, the TCGA dataset pertaining to cervical squamous cell carcinoma and endocervical adenocarcinoma (CESC) revealed no significant change in the expression of *EEF1A2* but a decrease in *EEF1A1* expression compared with normal tissue and GTEx normal samples (Fig. 4A, B). Moreover, examining the overall survival plot within the TCGA–CESC dataset unveiled a significant hazard ratio for both *EEF1A* isoforms in cervical cancer, as depicted in Fig. 4C. To gain a comprehensive grasp of the *EEF1A* isoforms expression patterns in cervical cancer, further investigations are imperative.

## Role of EEF1A2 in oncogenic processes

### Cellular proliferation

Ectopic *EEF1A2* expression enhanced the proliferative capacity of the SK-OV-3 ovarian carcinoma cell line [17]. Immortalized ovarian surface epithelial (IOSE) lines constitutively overexpressing *EEF1A2* exhibited enhanced proliferation potential over more extended periods than parental cell lines or controls. Interestingly, *EEF1A2* expressing lines proliferated better than their respective controls in low-serum conditions and even in the absence of serum [51]. In another study with mouse plasmacytomas, i.e., tumors of mature plasma cells, *EEF1A2* knockdown expression decreased IL-6-mediated activation of STAT3 and AKT pathways, leading to a decrease in proliferation of plasmacytomas (PCT) cell lines with delayed cell-cycle entry [6]. In a study conducted to explore the pro-tumorigenic role of EEF1A2 in prostate cancer, siRNA-mediated suppression of eEF1A2 protein resulted in reduced proliferation rate and colony formation in two prostate cancer cell lines, DU-145 and PC-3 [36]. Suppression of *EEF1A2* led to substantial upregulation of proteins associated with the apoptosis pathway, namely caspase-3, BAD, BAX, and PUMA. *EEF1A2* overexpression increases cell proliferation in the SW1990 pancreatic cancer cell line [42].

Overall, the research consistently demonstrates that *EEF1A2* expression promotes enhanced cell proliferation across various cancer types, suggesting its potential as a protumorigenic factor with implications for therapeutic targeting.

### Evasion of apoptosis

Escaping apoptosis is a major mechanism via which tumor cells remain viable and proliferate. Initial clues hinting at the anti-apoptotic role of eEF1A2 came from studies in myotubes, where EEF1A2 was found to exert a pro-survival effect, in stark contrast to EEF1A1, which exerted a pro-death activity [52]. Further evidence emerged upon discovering that EEF1A2 binds to Prdx-I, making the cells resistant to oxidative stress-induced cell death as a result. *EEF1A2* was subsequently found to support tumor cell survival via abrogation of apoptosis in HCC [53], mouse plasmacytomas [6], and prostate cancer [36]. Dependence on *EEF1A2* for resistance to apoptosis has also been documented in PA-1 ovarian cancer cells [51] and SH-SY5Y neuroblastoma cells [54].

These research findings strongly indicate that EEF1A2 plays a crucial role in promoting tumor cell survival by preventing apoptosis, as evidenced in multiple cancer types, and its binding to Prdx-I contributes to resistance against oxidative stress-induced cell death.

### Cellular migration and invasion

Over the past decade, a substantial body of evidence has been amassed, strongly indicating that EEF1A2 plays a crucial role in promoting the migratory and invasive characteristics of tumor cells. Amiri et al. [55] showed that ectopic expression of EEF1A2 enhanced cell migration and invasion in the TNBC line BT54 via activation of the PI3K–AKT signaling cascade in cytoskeletal remodeling. Likewise, the PI3K/AKT/NF-kB cascade was responsible for mediating an eEF1A2-associated increase in migratory and invasive properties of HCC cell lines [53]. siRNA-based knockdown of *EEF1A2* resulted in a reduction in migratory abilities of metastatic prostate cancer cell line PC3. *EEF1A2* imparted a more intense pro-migratory property to TNBC cell line MDA-MB-231 than MCF7 in an ERK-dependent manner [56]. *EEF1A2* could increase migration and invasion in neuroblastoma and glioblastoma cell lines in a PI3K-dependent manner [57].

Collectively, the evidence presented strongly supports the conclusion that *EEF1A2* plays a crucial role in promoting migratory and invasive characteristics of tumor cells through various signaling cascades, suggesting it as a potential target for therapeutic interventions in multiple cancer types.

### Interaction with other proteins

**Fig. 6:**
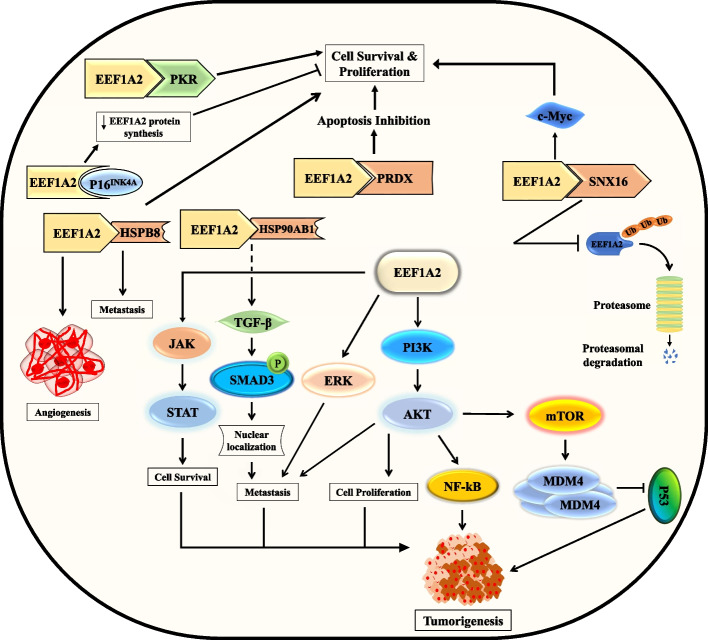
The oncogenic mechanisms of *EEF1A2*. Interacting partners of EEF1A2 and signaling pathway which regulate tumorigenesis. Arrows represent regulation of pathways and phenotype of tumor regulated through *EEF1A2*. T-shaped arrows indicate inhibition of targeted pathway

Panasyuk et al. [58] came up with an interesting hypothesis to explain the differential abilities of *EEF1A* isoforms in nontranslational functions. They showed that EEF1A2, but not EEF1A1, was able to interact with SH2 and SH3 domains of various signaling molecules. Hence, it could act as a more potent mediator of phosphotyrosine-mediated signaling. The ability of EEF1A2 to bind phosphatidylinositol-4 kinase β, leading to an increase in its lipid kinase activity and subsequent increase in phosphatidylinositol-4 phosphate production, weighs in on this role of EEF1A2 [9]. The exclusive ability of EEF1A2 to interact with other proteins became more evident when it was shown that *TSPY*, an oncogene, binds more strongly with EEF1A2 than EEF1A1. EEF1A2 has also been reported to enhance cell survival and proliferation via interaction with PRDX1, PKR, and SNX16 [59–61]. A recent study in LUAD showed that EEF1A2 interacts with HSP90AB1 and enhances TGFβ receptor leading to phosphorylation of SMAD3 expression and nuclear localization promoting EMT [37]. While these reports highlight the role of EEF1A2 in mediating oncogenic roles via interaction with protein partners, EEF1A2 itself has been reported to be affected by binding with other proteins. p16INK4a, a tumor suppressor protein belonging to the INK4 family of CDK inhibitors, was found to reduce the expression and function of *EEF1A2*, imparting its antiproliferative effects in the process [11] (Fig. 6).

The research by Panasyuk et al. and subsequent studies underscore the significant role of EEF1A2 in mediating cell survival and oncogenic processes through its distinct protein interactions, shedding light on its potential as a key player in various cellular pathways and highlighting its intricate interplay with other proteins [58].

### Activation of oncogenic pathways

Studies have frequently highlighted the role of the PI3K–AKT axis in mediating EEF1A2-mediated oncogenic effects in cells. Evidence of this effect came initially from the study of Amiri et al. [55] in breast cancer, which showed that EEF1A2 activated AKT and AKT-dependent actin remodeling for fueling migratory and invasive properties of the cells. The oncogenic effects of EEF1A2 in mice PCT were exerted via activation of JAK/STAT and AKT signaling [6]. EEF1A2 was found to enhance tumorigenic properties of HCC cell lines by stabilizing the oncogenic MDM4 protein via inactivation of p53 in a PI3K/AKT/mTOR-dependent manner [12]. *EEF1A2* knockdown in HCC cell lines in yet another study led to the abrogation of cancerous attributes of the tumor cells via the reduction of PI3K/AKT/NF-kB signaling [53]. SNX16 interaction with EEF1A2 triggers a c-Myc signaling cascade [61]. Our investigations have further elucidated the significant role of *EEF1A2* expression levels in neuroblastoma and glioblastoma cell lines. These levels exhibit a direct correlation with the cells proliferative, migratory, and invasive capacities, operating within the confines of a PI3K–AKT-dependent pathway [57]. Our team delved into the behavior of the triple-negative breast cancer cell line MDA-MB-231. Through the introduction of ectopic EEF1A2 expression, we observed a substantial augmentation in metastatic attributes. This effect was attributed to the activation of the ERK pathway [56].

Overall, *EEF1A2* plays a crucial role in mediating oncogenic effects in various cancer types, operating through the activation of multiple signaling pathways, including PI3K–AKT, JAK/STAT, and ERK, which promote tumor cell migration, invasion, and proliferation (Fig. 6).

## EEF1A2 as a therapeutic target

As aberrant overexpression of *EEF1A2* is an exclusive nature of cancerous cells, it presents itself as an attractive candidate for therapeutic intervention in cancers. The canonical function carried out by EEF1A2 in cells is redundant with the activities of the other *EEF1A* isoform, *EEF1A1*, which is also a promising candidate for targeting cancers. This is because EEF1A1 is an abundant cellular protein already present more than its partners in protein synthesis (molar ratios for EEF1A:EEF1B and EEF1A: ribosomes of 10:1 and 25:1, respectively) [62]. Thus, a 70% knockdown of *EEF1A1* itself will not have any deleterious effect on the regular translational activity of cells under nonstress conditions. Nonetheless, *EEF1A2* does not present such a situation. In recent years, multiple reports have emerged that have investigated the efficacy of selective targeting of *EEF1A2* in cancerous cells, and the results are pretty encouraging. Several reports utilizing shRNA or siRNA-based targeting of *EEF1A2* levels in cancerous cell lines have shown how depletion of *EEF1A2* compromises the oncogenic potential of tumor cells [53].

Interestingly, *EEF1A2* has been found to be a target of miR-663 and miR-744, which inhibit resveratrol-induced growth of MCF7 cells [20]. Most importantly, a marine natural product, plitidepsin, exerted its antitumor activity by targeting EEF1A2. What is more encouraging is that as of 2016, this drug has already successfully been in phase III clinical trials for multiple myeloma [22]. In a cancer-specific manner, *EEF1A2* has been reported to promote specific pathways, for example, TGF-β/SMAD signaling in lung adenocarcinoma [37], the PI3K/AKT/mTOR pathway in hepatocellular carcinoma [12], the ERK pathway in breast cancer [56], and PI3K signaling in brain cancer [57]. Targeting EEF1A2 with plitidepsin and cancer-specific pathway inhibitors [check https://www.cancer.gov/about-cancer/treatment/types/targeted-therapies/approved-drug-list for US Food and Drug Administration (FDA)-approved targeted therapy drugs] may provide a synergistic effect to inhibit cell survival and proliferation. Specific gene therapy targeting *EEF1A2* using clustered regularly interspaced short palindromic repeats (CRISPR) or an anti-*EEF1A2* peptide can be designed to target EEF1A2 interactions, which can hinder the tumorigenesis property of cancer.

In conclusion, targeting *EEF1A2* holds significant promise as a therapeutic strategy for cancer treatment. The progress in understanding the specific mechanisms and pathways associated with *EEF1A2* in different cancer types, coupled with the availability of targeted therapies such as plitidepsin, provides hope for more effective and precise cancer treatments in the future. Further research and clinical studies are warranted to explore the full potential of eEF1A2 as a target for cancer therapy, and to develop innovative and personalized treatments to combat this devastating disease.

## Discussion

The role of translation factors in cancers attracted the attention of researchers upon the discovery of the role of members of the proteins of the eukaryotic translational initiation machinery in different cancers. Among the elongation factors, members of the EEF1A protein family have garnered the most attention. Both EEF1A1 and EEF1A2 have been reported to be involved in mediating oncogenic processes, while the former has been associated with both pro- and anticancerous roles, *EEF1A2* has established itself as a potent oncogene in recent times. Table 1 presents compelling evidence for the role of *EEF1A2* as an oncogene across various cancer types. The majority of studies feature robust sample sizes of over 50 patients, with only two exceptions in the case of prostate cancer and one in ovarian cancer.

**Table 1:** EEF1A2 expression in cancer tissue and normal tissue, and its biological significance

Cancer type	EEF1A2 expression		Biological significance	References
	Cancer tissue (No. of samples)	Normal tissue (No. of samples)		
Ovarian cancer	High (159/500)	NA	No change in prognosis	[17]
	None, low, moderate (341/500)		Serous tumors with high EEF1A2 expression show good prognosis	
Ovarian cancer	High (3/148)	NA	NA	[32]
	Moderate (7/148)			
	Low (49/148)			
	None (89/148)			
Ovarian cancer	High (10/10) compared with benign endometriosis	NA	NA	[33]
Breast cancer	High (79/380)	NA	Good prognosis	[18]
	Moderate (177/380)		Good prognosis	
	Low (76/380)			
	None (48/380)			
Breast cancer	High (5/46)	Low (7/7)	NA	[34]
	Moderate (22/46)			
	Low (19/46)			
Triple-negative breast cancer	High (3/84)	NA	Poor prognosis	[35]
	Moderate (11/84)			
	Low (48/84)			
	None (22/84) compared with adjacent tissue			
Prostate cancer	High (3/4)	None (1/1)	NA	[14]
	None (1/4)			
Prostate cancer	High compared with adjacent normal tissue (26/30)	NA	NA	[36]
Prostate cancer	High (40/40) compared with benign tumor (8/8)	NA	Poor prognosis	[19]
Non-small cell lung cancer	High (58/69) compared with adjacent normal tissue of 46 patients	NA	Good prognosis	[38]
Lung adenocarcinoma	High compared with adjacent normal tissue (32/113)	NA	Poor prognosis	[16]
Lung adenocarcinoma	High compared with adjacent normal tissue (78/78)	NA	Poor prognosis	[37]
Pancreatic cancer	High (41/62)	NA	NA	[7]
Pancreatic ductal adenocarcinoma	High (76/97)	NA	Poor prognosis	[15]
Pancreatic cancer	High (29/35)	No expression (8/8)	NA	[42]
		Chronic pancreatitis shows no expression (13/13)		
Hepatocellular carcinoma	High (30/100)	None or very weak (27/27)	NA	[45]
	Moderate (24/100)	Adjacent normal tissue (84), high (1/84), moderate (14/84), weak (52/84), none (17/84)		
	Weak (29/100)			
	None (17/100)			
Hepatocellular carcinoma	High (24/48)	NA	Poor prognosis	[12]
	Low (24/48)	NA	Good prognosis	
Hepatocellular carcinoma	High (47/62)	None (20/20)	NA	[53]
Paired pericarcinomatous	High (5/62)			
Gastric cancer	High (105/129)	Weak (9/24)	Poor prognosis	[46]
		None (15/24)		

*NA* not available in the study

Although further research is required to conclusively establish EEF1A2 as a biomarker, its potential is promising, as multiple studies have demonstrated its association with cancer prognosis. It has been identified as an independent prognostic indicator for extended survival in serous cancer [17]. Additionally, it has been reported as a positive prognostic marker for breast cancer cases without lymph node involvement, and conversely, a negative prognostic marker for triple-negative breast cancer [18, 35]. In pancreatic cancer, its correlation with lymph node metastasis suggests its potential as an adverse prognostic marker [15]. In gastric cancer, the observed overexpression of *EEF1A2* has been established as an independent indicator for predicting poor prognosis [46]. Furthermore, in stage I non-small cell lung cancer patients, *EEF1A2* has been identified as a surrogate marker for poorer prognosis [16, 38].

In the context of prostate cancer, the overexpression of *EEF1A2* in metastatic prostate cancer suggests its potential as a tissue-based biomarker for monitoring cancer progression and cell transformation [19]. Additionally, in late-stage ovarian cancer, particularly in cases of serous endometrium cancer, the amplification of the 20q13 locus and *EEF1A2* copy number further underscore its potential as a biomarker for assessing patient survivability [3]. Moreover, elevated levels of *EEF1A2* in hepatocellular carcinoma biopsy samples suggest its potential as a diagnostic marker [12]. Collectively, these studies provide substantial evidence supporting the role of *EEF1A2* in cancer prognosis. Given these strong indications, it is imperative to conduct large-scale and systematic studies to firmly establish *EEF1A2* as a biomarker for various types of cancers.

We have identified notable disparities in *EEF1A2* expression across ovarian cancer, gastric cancer, and acute myeloid leukemia datasets derived from TCGA and individual reports. These discrepancies can be attributed to several factors. In the case of ovarian cancer, TCGA lacks information on normal tissue for comparison and does not distinguish between different subtypes, such as serous, endometrioid, mucinous, and clear cell. In contrast, scientific reports place emphasis on specific tissue types [17, 32]. Regarding acute myeloid leukemia, the study was conducted on cell lines (AML-193, Kasumi-1, and KG-1), rather than patient-derived cancer tissue. This difference in sample source may contribute to the observed disparity. Another potential reason for the inconsistency is that TCGA data is primarily RNA based, whereas other reports are based on protein expression data. Protein levels provide a more reliable indicator of functional implications in tumorigenesis. Additionally, the RNA data of TCGA relies on high-throughput platforms, which necessitates validation through RT–PCR. To address these discrepancies, an analysis of EEF1A2 protein expression within the same TCGA dataset at the protein level could offer valuable insights and potentially resolve the observed differences.

We have discussed the multiple facets of regulation mediated by *EEF1A2* in cancer progression. Unlike EEF1A1, EEF1A2 is able to interact with a number of binding partners and mediate the activation of key oncogenic pathways. The widely reported misexpression and redundant role of eEF1A2 in protein translation make eEF1A2 a very promising candidate for targeted therapy in cancers. We have discussed the multiple approaches in which this could be achieved. In this regard, dual targeting of both EEF1A isoforms could also be considered, since targeting of EEF1A1 isoform has also shown promising results in certain cancers such as HCC. The decision to target one or both of the EEF1A isoforms should be taken after consideration of the role of degree of pro-oncogenic role played by them in the specific tissue type [35], since we also see that *EEF1A2* could be associated with a better survival rate in certain cancers. Likewise, neoangiogenesis is essential for metastasis, and the role of *EEF1A2* in the regulation of the formation of a blood vessel in tumors needs urgent attention. *EEF1A2* targeting therapies could hold promise in a wide variety of cancer types. Plitidepsin-related clinical studies are still going on and are currently in the phase III stage for the treatment of lung and breast cancer, while it has successfully completed a phase III trial for multiple myeloma [22, 63].

## Conclusion

EEF1A proteins are not only involved in translation elongation process, but also in multiple noncanonical functions. The upregulation of EEF1A2 in various cancers such as ovarian, hepatocellular, breast, prostate, lung, and pancreatic, signifies its role in tumorigenesis. So far, the research has fully established the role of *EEF1A2* as an oncogene and in the regulation of JAK/STAT, ERK, PI3K/AKT/NF-kB, or mTOR oncogenic pathways. Numerous studies have highlighted the correlation between *EEF1A2* and cancer prognosis, suggesting its potential as a biomarker. Nonetheless, additional research is necessary to determine its accuracy and reliability as a biomarker within clinical settings. It is imperative to conduct further validations in larger and more diverse cohorts to firmly establish its potential utility. Furthermore, the specific upregulation of *EEF1A2* in cancers renders it an appealing target for drug development.

## Data Availability

Not applicable.

## Abbreviations

FISH: Fluorescence in situ hybridization
PCR: Polymerase chain reaction
EEF1A2: Eukaryotic translation elongation factor 1 alpha 2
EEF1A1: Eukaryotic translation elongation factor 1 alpha 1
UTR: Untranslated region
HCV: Hepatitis C virus
HBV: Hepatitis B virus
TMA: Tissue microarray
PCT: Plasmacytomas
